# Dietary Polyphenols in the Prevention of Stroke

**DOI:** 10.1155/2017/7467962

**Published:** 2017-10-24

**Authors:** A. Tressera-Rimbau, S. Arranz, M. Eder, A. Vallverdú-Queralt

**Affiliations:** ^1^Department of Nutrition, Food Science and Gastronomy, XaRTA, INSA-UB, School of Pharmacy and Food Science, University of Barcelona, Barcelona, Spain; ^2^CIBER Fisiopatología de la Obesidad y Nutrición, Instituto de Salud Carlos III, Madrid, Spain; ^3^AZTI Tecnalia Food Research Division, Derio, Bizkaia, Spain; ^4^INRA, UMR1083 Sciences pour l'œnologie, 2 Place Viala, 34060 Montpellier Cedex, France

## Abstract

Polyphenols have an important protective role against a number of diseases, such as atherosclerosis, brain dysfunction, stroke, cardiovascular diseases, and cancer. Cardiovascular diseases are the number one cause of death worldwide: more people die annually from cardiovascular diseases than from any other cause. The most important behavioural risk factors of heart disease and stroke are unhealthy diet, physical inactivity, tobacco use, and excess alcohol intake. The dietary consumption of polyphenols has shown to be inversely associated with morbidity and mortality by cardio- and cerebrovascular diseases. It is well-known that the protective effects of polyphenols *in vivo* depend on the grade how they are extracted from food and on their intestinal absorption, metabolism, and biological action with target tissues. The aim of this review was to summarise the relation between polyphenols of different plant sources and stroke in human intervention studies, animal models, and in vitro studies.

## 1. Introduction

Polyphenols are plant secondary metabolites and the most abundant dietary bioactive compounds. It is known that 100,000 to 200,000 secondary metabolites exist. Plant polyphenols include flavonoid and nonflavonoid compounds [1, 2]. Flavonoids are built from a basic structure made up of an oxygenated heterocycle and 2 phenolic rings. They are distinguished by the oxidation state of the heterocyclic pyran ring, forming several groups (e.g., flavonols, flavanols, and anthocyanins). Until now, over 4000 flavonoids have been described in plants [1], and this number is constantly expanding due to multiple ways by which primary substituents are replaced, yielding more complex structures. Moreover, flavan-3-ols are also found in oligomer and polymer forms, known as proanthocyanidins. The nonflavonoids include phenolic acids (benzoic and hydroxycinnamic acids) and stilbenes. Further nonflavonoids that may be found in nature are gallotannins, ellagitannins, stilbene oligomers, and lignans [2].

Polyphenols possess a wide range of beneficial effects against atherosclerosis, brain dysfunction, stroke, cardiovascular diseases (CVD), and cancer [1, 3]. The most important food sources are fruit and vegetables [3], red wine [4], black and green teas [5], coffee [6], extra virgin olive oil [7], and chocolate [8, 9]. Moreover, herbs, spices, and nuts are also potentially relevant polyphenol sources [10, 11]. The current dietary advice is that people should consume ≥5 portions of fruit and vegetables every day, each portion being at least 80 grams (U.S. Department of Health and Human Services, 2010), keep the body mass index from 18.5 to 24.9 kg/m^2^, and perform physical exercise [12].

It is well-known that the protective effects of polyphenols *in vivo* depend on the grade how they are extracted from food and on their intestinal absorption, metabolism, and biological action with target tissues [13]. In the human digestive system, polyphenols can be metabolized by intestine or hepatic cells or by intestinal microbiota. Advances in polyphenol bioavailability and metabolism, in the mechanisms of action, and in the evidence of health effects on animal models and humans have been reported [14]. There is an interindividual variability of polyphenol metabolites present in plasma and urine after the intake of different polyphenols which has been correlated with human gut microbiota composition and/or genetic polymorphisms, and it is potentially associated with specific health effects [15].

This review aims to summarise the current knowledge about the effects of dietary polyphenols from different food sources on stroke in human intervention studies, animal models, and *in vitro* studies.

## 2. Dietary Polyphenols and CVD

According to the reported information from the World Health Organization, CVD are the main cause of mortality worldwide. In 2012, CVD caused over 17.5 million deaths, which represented 31% of all deaths globally (7.4 and 6.7 million were caused by coronary heart disease and stroke, resp.). The main risk factors for CVD and stroke are lack of exercise, unhealthy diet, increased blood pressure, glucose and lipids, obesity, and extensive use of tobacco and alcohol. These risk factors should be controlled regularly and can be a sign of an increased risk for stroke, heart attack, and heart failure among others. Nonmodifiable risk factors for CVD are increasing age, gender, ethnicity, and family history. Thus, two-thirds of all strokes occur in people older than 65, mainly man and African-Caribbean.

Currently, the major risk factor for developing cardio- and cerebrovascular diseases is hypertension, and it is the major global health problem, affecting approximately 1 billion individuals and causing 7.6 million premature deaths, as well as 6% of all causes of disability worldwide [16]. Antihypertensives were the most distributed drugs (698 million prescriptions) in the US during 2013 [12].

The dietary consumption of polyphenols was shown to be inversely associated with cardio- and cerebrovascular diseases due to the anti-inflammatory and antiatherogenic properties of polyphenols, such as inhibition of peroxyl radical-induced DNA strand breakage, inhibition of platelet aggregation and of the expression of adhesion molecules to the endothelium, and protection of low-density lipoprotein (LDL) from oxidative damage [17, 18].

In certain inflammatory disease states, such as atherosclerosis, HDL particles may display atheroprotective functions in reverse cholesterol transport and antioxidant, anti-inflammatory, and antiapoptotic processes. Flavonoids show the potential to improve HDL function through their well-documented effects on cellular antioxidant status and inflammation [19]. Different clinical trials have revealed the atheroprotection of olive oil polyphenols through decreasing plasma LDL concentrations and LDL atherogenicity and improving HDL function [20, 21]. It has also been reported that polyphenols can reduce inflammation by blocking cytokines such as TNF-*α*, IL-1, IL-6, and IL-8 and C-reactive protein that play an important role as inflammatory markers in several diseases [18].

Several observational and short- or long-term intervention trials with food that possesses large amounts of polyphenols, such as virgin olive oil, cocoa, berries, and wine, have clearly shown that polyphenols have a positive effect on cardiovascular risk parameters. For example, BP levels are inversely correlated with the consumption of food rich in polyphenols. This may lead to the improvement in resistance artery function and decreases in peripheral artery vascular tone. However, most of these studies only take into account one sole source of polyphenols or one food item [22–27]. Noad et al. [28] demonstrated that a diet rich in polyphenols including six portions of fruits and vegetables, together with dark chocolate and a portion of berries each day, shows an improvement of microvascular function and decreases systolic BP. This is in accordance with several studies dealing with the vasodilator effects of polyphenols [29, 30]. Moreover, polyphenols can inhibit alpha-glucosidase and lipase activity [17].

Studies with wine and grape extracts have demonstrated improved endothelial function in conduit and resistance arteries [23, 26], possibly via nitric oxide- (NO-) dependent pathways [23]. In experimental studies, dietary polyphenols may help to stimulate NO endothelial secretion, which can decrease BP. Studies with tea and cocoa found an improvement in indirect measures of sympathetic nervous system activity patterns, possibly contributing to the lowering of peripheral vascular tone [27]. Moreover, hydroxytyrosol, oleuropein, and secoiridoids, found mainly in olives or virgin olive oils, may protect LDL particles from oxidation [12]. Further, cocoa flavanols have been assessed to play a role in maintaining endothelium-dependent vasodilation [12].

## 3. Dietary Polyphenols and Stroke

### 3.1. Stroke: Definition and Epidemiology

A stroke is a cerebrovascular disease related to atherosclerosis. It is caused by the interruption of the blood supply to the brain, usually because a vessel has either ruptured or been blocked by a blood clot. The result of this reduction in blood supply is a cut in the brain's oxygen causing the sudden death of some brain cells. Immediate symptoms are loss of speech, weakness, paralysis of one side of the body, dizziness, nausea, balance and coordination issues, and visual problems [31, 32].

Stroke is classified according to its aetiology as either haemorrhagic or ischaemic, being the last one as the most frequent (87% of cases). Haemorrhagic stroke is caused by a bleed into the brain tissue due to the rupture of blood vessels, aneurysms, or traumas. On the other hand, ischaemic stroke is caused by the occlusion of a cerebral artery, and it can be thrombotic or atherosclerotic and embolic or can be produced by microartery occlusion [31, 32].

Stroke is the second main cause of mortality worldwide and the main cause of adult disability, especially in middle- (12.8%) and high-income countries (8.7%). Ischaemic heart disease and stroke have been the world's biggest killers in the last 15 years. Only in 2015, they were together responsible of 15 million deaths around the world [33] and this burden is predicted to increase due to the rapid rise of population older than 65 years [31].

### 3.2. In Vitro Assays Dealing with Stroke Risk

Different mechanisms of polyphenols on stroke prevention have been elucidated through in vitro studies. A study carried out by Gundimeda et al. [34] showed that trace amounts of green tea polyphenol (GTPP) and epigallocatechin-3-gallate (EGCG) could exhibit neurite outgrowth-inhibiting activity and the growth cone-collapsing activity of Nogo-66 (C-terminal region of Nogo-A). Resveratrol and polyphenols from green tea were able to reduce the reactive oxygen species (ROS) in mitochondria and cell swelling in endothelial cells [35]. Recent evidence also showed that resveratrol can be considered a signaling molecule in tissues and cells and thus act as a modulator for the expression of genes and proteins [36]. Thus, they may prevent, in part, brain edema and neural damage. One possible mechanism is that polyphenols may decrease endothelial cell swelling through Ca^2+^ reduction.

Mangiferin and morin, two natural antioxidants from mango peel and *Maclura pomifera,* (commonly known as the Osage orange), respectively, exhibited a wide spectrum of antioxidant and antiapoptotic activities in an in vitro model at micromolar concentrations [37]. These polyphenols are promising neuroprotectors for the diverse pathologies, such as cerebrovascular accident, epilepsy, stroke, brain trauma, and spinal cord damage.

Spigoni et al. [38] observed in endothelial cells that urolithins A and B and urolithin *β*-glucuronide had an influence on NO and NO synthase activation. Urolithin metabolism may be linked to peripheral cells. eNOS expression was activated by a mix of urolithins at 5 *μ*M and urolithin *β*-glucuronide at 15 *μ*M. It is known that decreased eNOS activation and reduction in NO bioavailability are two of the key factors for endothelial dysfunction. Moreover, the mix of urolithins at 5 *μ*M following 24 h of incubation increased significantly nitrite/nitrate levels, also important in endothelial dysfunction.

Some reports show increased extracellular levels of glutamate in certain neurodegenerative diseases, such as stroke [39]. It is also very well-known that, in this brain disorder, increases in ROS levels occur, which have been associated with increased release and decreased uptake of glutamate [40]. Abib et al. [41] evaluated the effects of (−)-epicatechin-3-gallate (ECG), one of the three major green tea antioxidants, on C6 lineage cells. They observed that after 6 h, there was a beneficial cell response induced by ECG, indicating that ECG should be able to protect the brain against excitotoxicity induced by glutamate.

Another study reported that pycnogenol possesses scavenging activity against ROS and nitrogen species. In addition, effects on NO metabolism were proven in the macrophage cell line RAW 264.7 [42]. Cells were preincubated with pycnogenol, showing significant decreases in NO generation. This data sheds light on the biological activity of pycnogenol and possibly other polyphenols as beneficial agents in several human illnesses.

### 3.3. Animal Studies Determine the Molecular Mechanisms Involved in Stroke Prevention

Several epidemiological studies of dietary polyphenols on chronic disease prevention support a protective effect of these compounds against CVD [43–45]. Some observational studies have also shown an inverse association between the consumption of some classes of polyphenols and the overall mortality [44, 46]. Involved mechanisms have been elucidated from numerous studies conducted in animal models (mainly rodents) with nutritionally realistic levels of isolated flavonoids [47].

The health effect of polyphenols from tea, coffee, and cocoa has been extensively evaluated in experimental studies with animals, indicating beneficial effects on cardiovascular health and a reduced risk of stroke. While tea and cocoa exhibit beneficial effects on endothelial function, total and LDL cholesterol (tea only), and insulin sensitivity (cocoa only), moderate coffee consumption has been inversely associated with the risk of stroke. However, further long-term randomized clinical trials and prospective studies are necessary to provide clear mechanisms by which coffee, cocoa, and tea polyphenols offer cardiovascular health benefits. Antihypertensive, hypocholesterolemic, antioxidant, and anti-inflammatory activities have been proposed to these polyphenols, as well as improvement of vascular endothelial function and insulin sensitivity.

EGCG present in a limited number of plant-based foods and beverages, such as green tea, exhibited neuroprotective action in a cerebral ischaemia mouse model by controlling the inflammation cascade [48, 49], providing antioxidant and neuroprotective effects, possibly through the activation of the NF-E2-related factor 2/antioxidant responsive element signaling pathway [50]. A recent study described the first in vivo results supporting the potential of the coadministration of EGCG with a recombinant tissue plasminogen activator to extend its therapeutic window in treating acute brain ischaemia [51].

Quercetin, another flavonoid with similar antioxidant effects as those of green tea polyphenols, reduced levels of matrix metallopeptidase 9 (MMP-9) in cerebral ischaemia studies and attenuated blood-brain barrier disruption [52]. In a rat model of heat stroke, quercetin therapy (30 mg/kg) showed an improvement of heat stroke outcomes by attenuating excessive hyperthermia as well as myocardial injury. The protective effects of quercetin could be attributed to antilipid, peroxidative, antioxidant, and anti-inflammatory properties [53]. Antihypertensive effects in a rat model have also been attributed to quercetin that may induce a progressive and sustained reduction in BP, oxidative stress, or NO status [54]. Moreover, quercetin rutinoside (rutin) has been found to control neural damage in cerebral ischaemia [55].

Resveratrol has been extensively studied because of its ability to modulate certain parameters linked with increased cardiovascular risk [56]. Inhibition of lipid oxidation processes has been proposed as the main mechanism involved in neuroprotection. More recent results from a rat model of transient middle cerebral artery occlusion indicate that resveratrol significantly decreases apoptosis, mitochondrial lipid peroxidation, brain infarct volume, and edema [57, 58]. In the endothelium, resveratrol can stimulate NOS activity, increasing the amount of NO in isolated rat aortas [59].

Polyphenols from grape powder, administered as a supplement in a diet, have been reported to protect the brain against ischaemic damage. The neuroprotective effects of the GP supplement may have a wide implication in the future for the prevention/protection against other neurodegenerative damages [60].

Antioxidative, anti-inflammatory, antilipidemic, and neuroprotective properties have been attributed to curcumin in experimental animal models [61]. In a porcine model with endothelial dysfunction, for example, curcumin can effectively block the detrimental effect of homocysteine on the vascular system [62].

Diets enriched in anthocyanins from blueberries provide neuroprotection after stroke induced in rats due to their antiatherogenic and anti-inflammatory properties [63, 64]. Consumption of pomegranate byproduct resulted in a 57% reduction in atherosclerotic lesion size in mice [65]. A red wine extract, rich in anthocyanin, reduces injury induced by cerebral ischaemia in rats and protects against ischaemia-induced excitotoxicity, energy failure, and oxidative stress [66].

Hydroxytyrosol and oleuropein, polyphenols from olive oil, are recognized as having an important role in the protection against CVD [67]. Olive oil supplementation in rabbits improved the outcome of atherosclerosis by improving the lipid profile and reducing platelet hyperactivity [68–70].

### 3.4. Dietary Polyphenols and Stroke: Results from Human Clinical and Epidemiological Studies

Epidemiologic studies are a useful tool to assess the association between dietary intake of polyphenols and human health. Although no cause-effect information can be obtained from these studies, they have been broadly used to evaluate the effects of dietary compounds on several diseases. The inverse association between high-polyphenol content food (e.g., fruits, vegetables, and their derivatives) and CVD risk has been reported elsewhere and seems indisputable. However, the question as to whether polyphenols are responsible for this protection is still under debate [71–73].

The consumption of alcoholic beverages with a high polyphenol content (i.e., wine and beer) and stroke have also been studied. The results of different observational studies suggest that the relative risk of ischaemic stroke is lowered by moderate alcohol consumption [74]. In fact, it is generally accepted that the association between alcohol intake and the risk of CVD and total mortality is demonstrated by a J-shaped curve. The conclusion derived from these graphs is that having one or two drinks per day, without binge drinking, protects against CVD, particularly coronary disease and ischaemic stroke [75, 76].

Since the formulation of the concept of the French paradox by epidemiologists in the 1980s, many efforts have been made to elucidate its causes. The low incidence of CVD in the French population despite a high intake of saturated fats has been on the table for decades. Debates have focused on wine consumption since it is the common element in all southern European countries [77]. Red wine contains around 200 mg of polyphenols per 100 g [78] mainly anthocyanins and flavan-3-ols and is one of the main contributors to total polyphenol intake in these countries [79–81].

Many experimental studies with resveratrol and stroke have been conducted in animals; however, to date, no clinical investigations have been performed in humans who have survived a stroke. It is plausible that resveratrol increases cerebral blood flow and enhances cerebrovascular perfusion in these patients since these effects have been observed in healthy subjects [82]. Other *in vivo* and animal studies have reported benefits of wine polyphenols on stroke protection [54].

Wine consumption has frequently been associated with CVD protection although beer has been also studied. Nevertheless, only three studies have provided prospective data concerning beer consumption and stroke [83–85], and only one study found a significant inverse association for ischaemic stroke on comparing subjects who drank 1–6 drinks per week with abstainers (61%; 95% CI: 16% to 81%) [86]. Thus, the data on beer and stroke currently available are inconclusive albeit promising.

The effect of alcohol on cerebrovascular events is due to its capacity to improve the lipid profile (increasing HDL cholesterol and decreasing LDL cholesterol) and reduce platelet aggregation and its anti-inflammatory effects. Additionally, nonalcoholic components of wine and beer such as polyphenols exert antiatherogenic and antithrombotic effects and regulate endothelial function through different mechanisms [22].

Other polyphenol-rich drinks such as coffee or tea have been associated with a lower risk of stroke. As with alcohol, high consumption of coffee is considered a risk factor for CVD, but moderate consumption may reduce the risk. A meta-analysis of 11 prospective studies pointed out that the risk of stroke was lower in subjects who consumed 6 or less cups of coffee per day compared with nonconsumers. The relative risks were similar for ischaemic and haemorrhagic stroke, and no differences were observed with gender at lower levels of coffee consumption (2 cups per day or less) [87]. More recent prospective studies confirmed these results for stroke mortality and incidence [88–90]. It should be pointed out that the many different ways of coffee preparation can affect the polyphenol content and may lead to biased results. Moreover, the mechanisms by which coffee may exert these beneficial effects are not clear [87, 91].

Similar to coffee, teas are very rich in polyphenols, mainly flavonoids. It is known that tea flavonoids improve endothelial function, BP, cholesterol levels, and blood glucose concentrations [91, 92]. The results of a meta-analysis of 14 prospective studies showed that the increment of 3 cups/day in green or black tea consumption decreased the relative risk by 13% (95% confidence interval (CI), 0.81–0.94). The association did not depend on gender but was slightly higher for green tea. Other results from two large prospective studies of green and black tea were in line with these findings [90, 92].

Other typically polyphenol-rich foods are chocolate and cocoa, nuts, and olive oil. Prospective studies on chocolate and stroke are still scarce, although a significant reduction of 19% was observed in a meta-analysis of these studies when comparing the extreme of chocolate consumption [93].

Culinary herbs and spices are broadly used in meals to improve the flavour of food dishes and also as traditional medicines to prevent or treat different conditions. Scientific evidence about this is still scarce, but the results of the studies available are promising. Polyphenols may be found among all the active chemical components of herbs and spices. Due to the lack of water, the concentration of polyphenols in herbs and spices is higher than in fruits and vegetables. Several studies have focused on the effects of herbs, spices, and medicinal plants on glucose regulation and dyslipidemia in subjects who had metabolic syndrome and type 2 diabetes, both of which are major risk factors for stroke [94]. However, to our knowledge, no clinical trials or clinical studies have been performed to evaluate the direct relationship between spices/herbs and the risk of stroke.

Novel meta-analyses reported the effects of flavanol-containing products such as tea, cocoa, and apple products on body composition, blood pressure, and blood lipids. Bertoia et al. [95] indicated that foods rich in flavan-3-ols, flavonols, anthocyanins, and flavonoid polymers may help adults to control body weight. This type of studies may give a dietary pattern for obesity prevention and its possible consequences.

Several meta-analyses of randomized controlled trials have dealt with the effect of tea and different outcomes. The effects of black tea on blood cholesterol are contradictory. One meta-analysis found no effects of black tea on total cholesterol and serum concentrations of LDL and HDL cholesterol [96], while another one concluded that consumption of black tea lowered LDL cholesterol, especially in subjects at high cardiovascular risk [97]. Acute black tea and chocolate increased flow-mediated dilatation after acute and chronic intake and reduced systolic and diastolic blood pressure. Regarding green tea, results from randomized controlled trials point in the same direction. Its intake resulted in significant reductions in systolic blood pressure, total cholesterol, and LDL cholesterol [98–100].

Several randomized controlled trials have been conducted to assess the effects of cocoa and dark chocolate, which are rich sources of flavonoids, on serum lipids. Total cholesterol and LDL cholesterol decreased when consuming these foods. No effects were observed for HDL cholesterol and triglyceride concentration in plasma [101]. However, the observed changes depend on the intake dose and the health of the participants [102].

The PREDIMED (*Prevención con Dieta Mediterránea*) study was a prospective, randomized, multicentre, controlled trial aimed at determining the benefits of following a Mediterranean diet supplemented with either nuts or extra virgin olive oil on cardiovascular events (including myocardial infarction, stroke, and cardiovascular death). The control group followed a low-fat diet according to the guidelines of the American Heart Association. At the end of the study, the results revealed that both Mediterranean diet groups had a 30% lower incidence of the primary endpoint than the control group. Remarkably, incident stroke was reduced by nearly 50% in participants following the Mediterranean diet enriched with nuts (15 g walnuts, 7.5 g almonds, and 7.5 g hazelnuts) [103].

In an observational study within the PREDIMED trial, total polyphenol intake and polyphenol subclasses were calculated from the FFQ and the Phenol-Explorer database. Total polyphenols were associated with a 46% reduction in CVD risk on comparing the fifth versus the first quintiles. The intake of lignans, flavanols, and hydroxybenzoic acids was also inversely and significantly associated with cardiovascular events [104]. Besides this study, only a few more have directly evaluated the association between polyphenol intake and stroke, and only 2 found a significant inverse association: the Zutphen Elderly Study and the Finnish Mobile Clinic Health Examination Survey. In the first study, flavonol and flavone intakes, but not catechin intake [46], were associated with a lower incidence of stroke [105]. In the Finnish cohort, the polyphenolic groups inversely associated with stroke were flavonols, flavones, and flavanones [106]. However, similar studies did not find any association [107, 108].

## 4. Mechanisms

**Figure 1:**
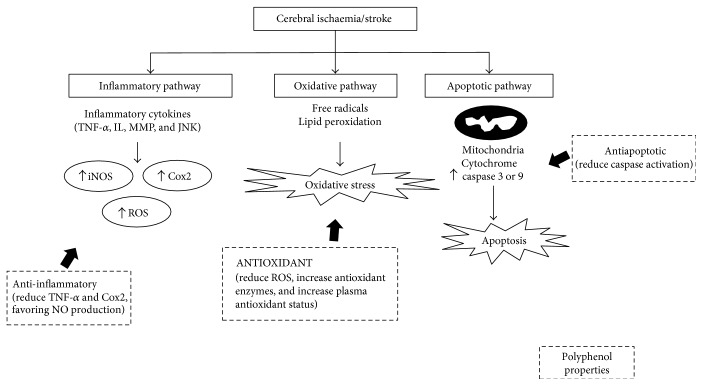
Molecular signaling pathways and molecular risk factors modulated by polyphenols in neurological disorders.

Polyphenols from fruits, vegetables, and beverages, such as wine, tea, cocoa, may exert protective effects on the cardiovascular system [109]. Increasing evidence suggests that dietary polyphenols prompt their health properties by the interaction with molecular signaling pathways [110] (Figure 1). The ability of polyphenols to target transcriptional networks that modulate gene expression favoring NO production, anti-inflammatory mediators, and energy expenses provides an attractive pharmacological approach to treat cardiovascular and metabolic diseases.

One of the most well-described mechanisms on neurovascular protection involves the ability of polyphenols to generate NO, a potent vasodilator generated by endothelial cells, which can also regulate cardiovascular-related gene expression. Consequently, polyphenols prevent the progress of endothelial dysfunction, decreasing the development of atherosclerotic plaque, vascular thrombosis, and occlusion. In early stages of atherosclerosis development, polyphenols contribute to reducing LDL oxidation, improving antioxidant status, and decreasing levels of inflammatory cytokines and adhesion molecules (Figure 1).

Oxidative stress is thought to be a key event in the pathogenesis of cerebral ischaemia. Overproduction of ROS during ischaemia may cause an imbalance between oxidative and antioxidative processes. ROS can damage lipids, proteins, and nucleic acids, inducing apoptosis or necrosis. It is hypothesized that plant polyphenols can provide protection against neurodegenerative changes associated with cerebral ischaemia [56]. Polyphenols can upregulate multiple redox enzymes, such as endothelial NO synthase, catalase, SOD1, and SOD2.

Most experimental and epidemiological studies suggest that dietary polyphenols activate antioxidant pathways and modulate immune response by inhibiting proinflammatory biomarkers.

## 5. Conclusion

The results from experimental *in vitro* and *in vivo* studies and clinical evidence support the beneficial effects of some classes of polyphenols, mainly flavonoids, on multiple endpoints of cardiovascular risk. The most convincing mechanisms involve the reduction of BP by increasing NO production and improvement in endothelial function. However, the lack of information about polyphenolic food composition and metabolism does not allow conclusions about the effects and effectiveness in preventing CVD.

Moreover, future clinical trials evaluating the effect of dietary polyphenols should take into account different aspects to obtain conclusive results. It is important to include a proper control product regarding polyphenol content. It is also necessary to consider the specific target population included in the study to correctly design the nutritional intervention period duration (weeks, months), as well as the dose of the polyphenols investigated. To establish the effects of oral exposure to the most promising polyphenols, large-scale, long-term, well-controlled trials, preferably with clinical endpoints including cardiovascular morbidity/mortality, are required. Until now, there is no sufficient data to establish a dietary reference intake for each class of polyphenols. To ensure the safety of their consumption, the development of functional foods should only consider the same level of polyphenols as the best dietary source can deliver at dietary doses. Then, the bioactivity of this functional food should be tested with *in vivo* models. After proving the bioefficacy of the compounds, mechanism should be tested *in vitro.*

In the future, it is essential for the scientific community to strictly consider well-designed experiments (in terms of the nature and concentrations of metabolites used) in order to definitively identify the compounds biologically active in target tissues as well as the molecular mechanisms involved.

## References

[1] Del Rio D., Rodriguez-Mateos A., Spencer J. P. E., Tognolini M., Borges G., Crozier A. (2013). *Dietary (Poly)phenolics in Human Health: Structures, Bioavailability, and Evidence of Protective Effects against Chronic Diseases*.

[2] Lamuela-Raventós R. M., Vallverdú-Queralt A., Jáuregui O., Martínez-Huélamo M., Quifer-Rada P. (2014). Improved characterization of polyphenols using liquid chromatography. *Polyphenols in Plants*.

[3] Crozier A., Jaganath I. B., Clifford M. N. (2009). Dietary phenolics: chemistry, bioavailability and effects on health. *Natural Product Reports*.

[4] Vallverdú-Queralt A., Boix N., Piqué E. (2015). Identification of phenolic compounds in red wine extract samples and zebrafish embryos by HPLC-ESI-LTQ-Orbitrap-MS. *Food Chemistry*.

[5] Frei B., Higdon J. V. (2003). Antioxidant activity of tea polyphenols in vivo: evidence from animal studies. *The Journal of Nutrition*.

[6] Bonita J. S., Mandarano M., Shuta D., Vinson J. (2007). Coffee and cardiovascular disease: in vitro, cellular, animal, and human studies. *Pharmacological Research*.

[7] Talhaoui N., Gómez-Caravaca A. M., León L., De la Rosa R., Fernández-Gutiérrez A., Segura-Carretero A. (2016). From olive fruits to olive oil: phenolic compound transfer in six different olive cultivars grown under the same agronomical conditions. *International Journal of Molecular Sciences*.

[8] Visioli F., Bernaert H., Corti R. (2009). Chocolate, lifestyle, and health. *Critical Reviews in Food Science and Nutrition*.

[9] Rusconi M., Conti A. (2010). *Theobroma cacao* L., the food of the gods: a scientific approach beyond myths and claims. *Pharmacological Research*.

[10] Vallverdú-Queralt A., Regueiro J., Martínez-Huélamo M., Alvarenga J. F. R., Leal L. N., Lamuela-Raventos R. M. (2014). A comprehensive study on the phenolic profile of widely used culinary herbs and spices: rosemary, thyme, oregano, cinnamon, cumin and bay. *Food Chemistry*.

[11] Regueiro J., Sánchez-González C., Vallverdú-Queralt A., Simal-Gándara J., Lamuela-Raventós R., Izquierdo-Pulido M. (2014). Comprehensive identification of walnut polyphenols by liquid chromatography coupled to linear ion trap–Orbitrap mass spectrometry. *Food Chemistry*.

[12] Lamuela-Raventos R. M., Quifer-Rada P. (2016). Effect of dietary polyphenols on cardiovascular risk. *Hear*.

[13] Martínez-Huélamo M., Vallverdú-Queralt A., Di Lecce G. (2016). Bioavailability of tomato polyphenols is enhanced by processing and fat addition: evidence from a randomized feeding trial. *Molecular Nutrition & Food Research*.

[14] Visioli F., De La Lastra C. A., Andres-Lacueva C. (2011). Polyphenols and human health: a prospectus. *Critical Reviews in Food Science and Nutrition*.

[15] Bolca S., Van de Wiele T., Possemiers S. (2013). Gut metabotypes govern health effects of dietary polyphenols. *Current Opinion in Biotechnology*.

[16] Medina-Remon A., Estruch R., Tresserra-Rimbau A., Vallverdú-Queralt A., Lamuela-Raventos R. M. (2013). The effect of polyphenol consumption on blood pressure. *Mini-Reviews in Medicinal Chemistry*.

[17] de Camargo A. C., Regitano-d’Arce M. A. B., Biasoto A. C. T., Shahidi F. (2014). Low molecular weight phenolics of grape juice and winemaking byproducts: antioxidant activities and inhibition of oxidation of human low-density lipoprotein cholesterol and DNA strand breakage. *Journal of Agricultural and Food Chemistry*.

[18] Denny C., Lazarini J. G., Franchin M. (2014). Bioprospection of Petit Verdot grape pomace as a source of anti-inflammatory compounds. *Journal of Functional Foods*.

[19] Millar C. L., Duclos Q., Blesso C. N. (2017). Effects of dietary flavonoids on reverse cholesterol transport, HDL metabolism, and HDL function. *Advances in Nutrition: An International Review Journal*.

[20] Hernáez Á., Fernández-Castillejo S., Farràs M. (2014). Olive oil polyphenols enhance high-density lipoprotein function in humans: a randomized controlled trial. *Arteriosclerosis, Thrombosis, and Vascular Biology*.

[21] Hernaez A., Remaley A. T., Farras M. (2015). Olive oil polyphenols decrease LDL concentrations and LDL atherogenicity in men in a randomized controlled trial. *Journal of Nutrition*.

[22] Chiva-Blanch G., Magraner E., Condines X. (2015). Effects of alcohol and polyphenols from beer on atherosclerotic biomarkers in high cardiovascular risk men: a randomized feeding trial. *Nutrition, Metabolism, and Cardiovascular Diseases*.

[23] Botden I. P. G., Langendonk J. G., Meima M. E. (2011). Daily red wine consumption improves vascular function by a soluble guanylyl cyclase-dependent pathway. *American Journal of Hypertension*.

[24] Botden I. P., Draijer R., Westerhof B. E. (2012). Red wine polyphenols do not lower peripheral or central blood pressure in high normal blood pressure and hypertension. *American Journal of Hypertension*.

[25] Droste D. W., Iliescu C., Vaillant M. (2013). A daily glass of red wine associated with lifestyle changes independently improves blood lipids in patients with carotid arteriosclerosis: results from a randomized controlled trial. *Nutrition Journal*.

[26] Siasos G., Tousoulis D., Kokkou E. (2013). Favorable effects of concord grape juice on endothelial function and arterial stiffness in healthy smokers. *American Journal of Hypertension*.

[27] Steptoe A., Gibson E. L., Vuononvirta R. (2007). The effects of tea on psychophysiological stress responsivity and post-stress recovery: a randomised double-blind trial. *Psychopharmacology*.

[28] Noad R. L., Rooney C., McCall D. (2016). Beneficial effect of a polyphenol-rich diet on cardiovascular risk: a randomised control trial. *Heart*.

[29] Medina-Remón A., Tresserra-Rimbau A., Pons A. (2015). Effects of total dietary polyphenols on plasma nitric oxide and blood pressure in a high cardiovascular risk cohort. The PREDIMED randomized trial. *Nutrition, Metabolism and Cardiovascular Diseases*.

[30] Guo X., Tresserra-Rimbau A., Estruch R. (2016). Effects of polyphenol, measured by a biomarker of total polyphenols in urine, on cardiovascular risk factors after a long-term follow-up in the PREDIMED study. *Oxidative Medicine and Cellular Longevity*.

[31] Abbott H., Sim F. (2010). *Unit: Public Health Aspects of Stroke Workbook*.

[32] Wittenauer R., Smith L. (2012). *Priority Medicines for Europe and the World “A Public Health Approach to Innovation”. Background Paper 6.6. Ischaemic and Haemorrhagic Stroke*.

[33] The World Health Organization (2017). *The Top 10 Causes of Death*.

[34] Gundimeda U., McNeill T. H., Barseghian B. A. (2015). Polyphenols from green tea prevent antineuritogenic action of Nogo-A via 67-kDa laminin receptor and hydrogen peroxide. *Journal of Neurochemistry*.

[35] Panickar K. S., Qin B., Anderson R. A. (2015). Ischemia-induced endothelial cell swelling and mitochondrial dysfunction are attenuated by cinnamtannin D1, green tea extract, and resveratrol in vitro. *Nutritional Neuroscience*.

[36] Doré S. (2005). Unique properties of polyphenol stilbenes in the brain: more than direct antioxidant actions; gene/protein regulatory activity. *Neurosignals*.

[37] Campos-Esparza M. R., Sánchez-Gómez M. V., Matute C. (2009). Molecular mechanisms of neuroprotection by two natural antioxidant polyphenols. *Cell Calcium*.

[38] Spigoni V., Mena P., Cito M. (2016). Effects on nitric oxide production of urolithins, gut-derived ellagitannin metabolites, in human aortic endothelial cells. *Molecules*.

[39] Campiani G., Fattorusso C., De Angelis M. (2003). Neuronal high-affinity sodium-dependent glutamate transporters (EAATs): targets for the development of novel therapeutics against neurodegenerative diseases. *Current Pharmaceutical Design*.

[40] Bowling A. C., Beal M. F. (1995). Bioenergetic and oxidative stress in neurodegenerative diseases. *Life Sciences*.

[41] Abib R. T., Quincozes-Santos A., Nardin P. (2008). Epicatechin gallate increases glutamate uptake and S100B secretion in C6 cell lineage. *Molecular and Cellular Biochemistry*.

[42] Virgili F., Kobuchi H., Packer L. (1998). Procyanidins extracted from *Pinus maritima* (Pycnogenol®): scavengers of free radical species and modulators of nitrogen monoxide metabolism in activated murine RAW 264.7 macrophages. *Free Radical Biology & Medicine*.

[43] Kuriyama S., Shimazu T., Ohmori K. (2006). Green tea consumption and mortality due to cardiovascular disease, cancer, and all causes in Japan: the Ohsaki study. *JAMA*.

[44] Mink P. J., Scrafford C. G., Barraj L. M. (2007). Flavonoid intake and cardiovascular disease mortality: a prospective study in postmenopausal women. *The American Journal of Clinical Nutrition*.

[45] Szmitko P. E., Verma S. (2005). Cardiology patient pages. Red wine and your heart. *Circulation*.

[46] Arts I. C., Hollman P. C., Feskens E. J., Bueno de Mesquita H. B., Kromhout D. (2001). Catechin intake might explain the inverse relation between tea consumption and ischemic heart disease: the Zutphen Elderly Study. *The American Journal of Clinical Nutrition*.

[47] Norata G. D., Marchesi P., Passamonti S., Pirillo A., Violi F., Catapano A. L. (2007). Anti-inflammatory and anti-atherogenic effects of cathechin, caffeic acid and trans-resveratrol in apolipoprotein E deficient mice. *Atherosclerosis*.

[48] Park J.-W., Hong J.-S., Lee K.-S., Kim H.-Y., Lee J.-J., Lee S.-R. (2010). Green tea polyphenol (−)-epigallocatechin gallate reduces matrix metalloproteinase-9 activity following transient focal cerebral ischemia. *The Journal of Nutritional Biochemistry*.

[49] Ashafaq M., Raza S. S., Khan M. M. (2012). Catechin hydrate ameliorates redox imbalance and limits inflammatory response in focal cerebral ischemia. *Neurochemical Research*.

[50] Han J., Wang M., Jing X., Shi H., Ren M., Lou H. (2014). (−)-Epigallocatechin gallate protects against cerebral ischemia-induced oxidative stress via Nrf2/ARE signaling. *Neurochemical Research*.

[51] You Y.-P. (2016). Epigallocatechin gallate extends the therapeutic window of recombinant tissue plasminogen activator treatment in ischemic rats. *Journal of Stroke and Cerebrovascular Diseases*.

[52] Lee J.-K., Kwak H.-J., Piao M.-S., Jang J.-W., Kim S.-H., Kim H.-S. (2011). Quercetin reduces the elevated matrix metalloproteinases-9 level and improves functional outcome after cerebral focal ischemia in rats. *Acta Neurochirurgica*.

[53] Lin X., Lin C.-H., Zhao T. (2017). Quercetin protects against heat stroke-induced myocardial injury in male rats: antioxidative and antiinflammatory mechanisms. *Chemico-Biological Interactions*.

[54] Perez-Vizcaino F., Duarte J., Andriantsitohaina R. (2006). Endothelial function and cardiovascular disease: effects of quercetin and wine polyphenols. *Free Radical Research*.

[55] Khan M. M., Ahmad A., Ishrat T. (2009). Rutin protects the neural damage induced by transient focal ischemia in rats. *Brain Research*.

[56] Simonyi A., Wang Q., Miller R. (2005). Polyphenols in cerebral ischemia: novel targets for neuroprotection. *Molecular Neurobiology*.

[57] Yousuf S., Atif F., Ahmad M. (2009). Resveratrol exerts its neuroprotective effect by modulating mitochondrial dysfunctions and associated cell death during cerebral ischemia. *Brain Research*.

[58] Baur J. A., Sinclair D. A. (2006). Therapeutic potential of resveratrol: the in vivo evidence. *Nature Reviews Drug Discovery*.

[59] Wallerath T., Deckert G., Ternes T. (2002). Resveratrol, a polyphenolic phytoalexin present in red wine, enhances expression and activity of endothelial nitric oxide synthase. *Circulation*.

[60] Wang Q., Simonyi A., Li W. (2005). Dietary grape supplement ameliorates cerebral ischemia-induced neuronal death in gerbils. *Molecular Nutrition & Food Research*.

[61] Ramirez-Tortosa M. C., Mesa M. D., Aguilera M. C. (1999). Oral administration of a turmeric extract inhibits LDL oxidation and has hypocholesterolemic effects in rabbits with experimental atherosclerosis. *Atherosclerosis*.

[62] Ramaswami G., Chai H., Yao Q., Lin P. H., Lumsden A. B., Chen C. (2004). Curcumin blocks homocysteine-induced endothelial dysfunction in porcine coronary arteries. *Journal of Vascular Surgery*.

[63] Del Rio D., Borges G., Crozier A. (2010). Berry flavonoids and phenolics: bioavailability and evidence of protective effects. *British Journal of Nutrition*.

[64] Basu A., Rhone M., Lyons T. J. (2010). Berries: emerging impact on cardiovascular health. *Nutrition Reviews*.

[65] Wu X., Kang J., Xie C. (2010). Dietary blueberries attenuate atherosclerosis in apolipoprotein E-deficient mice by upregulating antioxidant enzyme expression. *Journal of Nutrition*.

[66] Ritz M.-F., Curin Y., Mendelowitsch A., Andriantsitohaina R. (2008). Acute treatment with red wine polyphenols protects from ischemia-induced excitotoxicity, energy failure and oxidative stress in rats. *Brain Research*.

[67] Covas M.-I., de la Torre R., Fito M. (2015). Virgin olive oil: a key food for cardiovascular risk protection. *British Journal of Nutrition*.

[68] De La Cruz J. P., Villalobos M. A., Carmona J. A., Martin-Romero M., Smith-Agreda J. M., de la Cuesta F. S. (2000). Antithrombotic potential of olive oil administration in rabbits with elevated cholesterol. *Thrombosis Research*.

[69] Gonzalez-Santiago M., Martin-Bautista E., Carrero J. J. (2006). One-month administration of hydroxytyrosol, a phenolic antioxidant present in olive oil, to hyperlipemic rabbits improves blood lipid profile, antioxidant status and reduces atherosclerosis development. *Atherosclerosis*.

[70] Manna C., Migliardi V., Golino P. (2004). Oleuropein prevents oxidative myocardial injury induced by ischemia and reperfusion. *The Journal of Nutritional Biochemistry*.

[71] Arts I. C. W., Hollman P. C. H. (2005). Polyphenols and disease risk in epidemiologic studies. *The American Journal of Clinical Nutrition*.

[72] He F. J., Nowson C. A., MacGregor G. A. (2006). Fruit and vegetable consumption and stroke: meta-analysis of cohort studies. *The Lancet*.

[73] Saita E., Kondo K., Momiyama Y. (2015). Anti-inflammatory diet for atherosclerosis and coronary artery disease: antioxidant foods. *Clinical Medicine Insights: Cardiology*.

[74] Pinder R. M., Sandler M. (2004). Alcohol, wine and mental health: focus on dementia and stroke. *Journal of Psychopharmacology*.

[75] de Gaetano G., Costanzo S., Di Castelnuovo A. (2016). Effects of moderate beer consumption on health and disease: a consensus document. *Nutrition, Metabolism and Cardiovascular Diseases*.

[76] Di Castelnuovo A., Costanzo S., di Giuseppe R., de Gaetano G., Iacoviello L. (2009). Alcohol consumption and cardiovascular risk: mechanisms of action and epidemiologic perspectives. *Future Cardiology*.

[77] Ferrières J. (2004). The French paradox: lessons for other countries. *Heart*.

[78] Neveu V., Perez-Jiménez J., Vos F. (2010). Phenol-Explorer: an online comprehensive database on polyphenol contents in foods. *Database*.

[79] Tresserra-Rimbau A., Medina-Remón A., Pérez-Jiménez J. (2013). Dietary intake and major food sources of polyphenols in a Spanish population at high cardiovascular risk: the PREDIMED study. *Nutrition, Metabolism and Cardiovascular Diseases*.

[80] Pérez-Jiménez J., Fezeu L., Touvier M. (2011). Dietary intake of 337 polyphenols in French adults. *The American Journal of Clinical Nutrition*.

[81] Godos J., Marventano S., Mistretta A., Galvano F., Grosso G. (2017). Dietary sources of polyphenols in the Mediterranean healthy eating, aging and lifestyle (MEAL) study cohort. *International Journal of Food Sciences and Nutrition*.

[82] Bonnefont-Rousselot D. (2016). Resveratrol and cardiovascular diseases. *Nutrients*.

[83] Truelsen T., Gronbaek M., Schnohr P., Boysen G. (1998). Intake of beer, wine, and spirits and risk of stroke: the Copenhagen City Heart Study. *Stroke*.

[84] Djoussé L., Himali J. J., Beiser A., Kelly-Hayes M., Wolf P. A. (2009). Apolipoprotein E, alcohol consumption, and risk of ischemic stroke: the Framingham Heart Study revisited. *Journal of Stroke and Cerebrovascular Diseases*.

[85] Mukamal K. J., Ascherio A., Mittleman M. A. (2005). Alcohol and risk for ischemic stroke in men: the role of drinking patterns and usual beverage. *Annals of Internal Medicine*.

[86] Mukamal K. J., Chung H., Jenny N. S. (2005). Alcohol use and risk of ischemic stroke among older adults: the Cardiovascular Health Study. *Stroke*.

[87] Larsson S. C., Orsini N. (2011). Coffee consumption and risk of stroke: a dose-response meta-analysis of prospective studies. *American Journal of Epidemiology*.

[88] Freedman N. D., Park Y., Abnet C. C., Hollenbeck A. R., Sinha R. (2012). Association of coffee drinking with total and cause-specific mortality. *The New England Journal of Medicine*.

[89] Floegel A., Pischon T., Bergmann M. M., Teucher B., Kaaks R., Boeing H. (2012). Coffee consumption and risk of chronic disease in the European Prospective Investigation into Cancer and Nutrition (EPIC)-Germany study. *The American Journal of Clinical Nutrition*.

[90] Kokubo Y., Iso H., Saito I. (2013). The impact of green tea and coffee consumption on the reduced risk of stroke incidence in Japanese population: the Japan public health center-based study cohort. *Stroke*.

[91] Larsson S. C. (2014). Coffee, tea, and cocoa and risk of stroke. *Stroke*.

[92] Larsson S. C., Virtamo J., Wolk A. (2013). Black tea consumption and risk of stroke in women and men. *Annals of Epidemiology*.

[93] Larsson S. C., Virtamo J., Wolk A. (2012). Chocolate consumption and risk of stroke: a prospective cohort of men and meta-analysis. *Neurology*.

[94] Panickar K. S. (2013). Beneficial effects of herbs, spices and medicinal plants on the metabolic syndrome, brain and cognitive function. *Central Nervous System Agents in Medicinal Chemistry*.

[95] Bertoia M. L., Rimm E. B., Mukamal K. J., Hu F. B., Willett W. C., Cassidy A. (2016). Dietary flavonoid intake and weight maintenance: three prospective cohorts of 124,086 US men and women followed for up to 24 years. *BMJ*.

[96] Wang D., Chen C., Wang Y., Liu J., Lin R. (2014). Effect of black tea consumption on blood cholesterol: a meta-analysis of 15 randomized controlled trials. *PLoS One*.

[97] Zhao Y., Asimi S., Wu K., Zheng J., Li D. (2015). Black tea consumption and serum cholesterol concentration: systematic review and meta-analysis of randomized controlled trials. *Clinical Nutrition*.

[98] Khalesi S., Sun J., Buys N., Jamshidi A., Nikbakht-Nasrabadi E., Khosravi-Boroujeni H. (2014). Green tea catechins and blood pressure: a systematic review and meta-analysis of randomised controlled trials. *European Journal of Nutrition*.

[99] Kim A., Chiu A., Barone M. K. (2011). Green tea catechins decrease total and low-density lipoprotein cholesterol: a systematic review and meta-analysis. *Journal of the American Dietetic Association*.

[100] Zheng X.-X., Y-L X., Li S.-H., Liu X.-X., Hui R., Huang X.-H. (2011). Green tea intake lowers fasting serum total and LDL cholesterol in adults: a meta-analysis of 14 randomized controlled trials. *The American Journal of Clinical Nutrition*.

[101] Tokede O. A., Gaziano J. M., Djousse L. (2011). Effects of cocoa products/dark chocolate on serum lipids: a meta-analysis. *European Journal of Clinical Nutrition*.

[102] Jia L., Liu X., Bai Y. Y. (2010). Short-term effect of cocoa product consumption on lipid profile: a meta-analysis of randomized controlled trials. *The American Journal of Clinical Nutrition*.

[103] Estruch R., Ros E., Salas-Salvadó J. (2013). Primary prevention of cardiovascular disease with a Mediterranean diet. *New England Journal of Medicine*.

[104] Tresserra-Rimbau A., Rimm E. B., Medina-Remón A. (2014). Inverse association between habitual polyphenol intake and incidence of cardiovascular events in the PREDIMED study. *Nutrition, Metabolism, and Cardiovascular Diseases*.

[105] Keli S. O., Hertog M. G., Feskens E. J., Kromhout D. (1996). Dietary flavonoids, antioxidant vitamins, and incidence of stroke: the Zutphen study. *Archives of Internal Medicine*.

[106] Knekt P., Kumpulainen J., Järvinen R. (2002). Flavonoid intake and risk of chronic diseases. *The American Journal of Clinical Nutrition*.

[107] Hirvonen T., Virtamo J., Korhonen P., Albanes D., Pietinen P. (2000). Intake of flavonoids, carotenoids, vitamins C and E, and risk of stroke in male smokers. *Stroke*.

[108] Yochum L., Kushi L. H., Meyer K., Folsom A. R. (1999). Dietary flavonoid intake and risk of cardiovascular disease in postmenopausal women. *American Journal of Epidemiology*.

[109] Curin Y., Andriantsitohaina R. (2005). Polyphenols as potential therapeutical agents against cardiovascular diseases. *Pharmacological Reports*.

[110] Gonzalez-Gallego J., Garcia-Mediavilla M. V., Sanchez-Campos S., Tunon M. J. (2010). Fruit polyphenols, immunity and inflammation. *British Journal of Nutrition*.

